# Increased risk of high-grade prostate cancer among testicular cancer survivors

**DOI:** 10.1371/journal.pone.0263573

**Published:** 2022-02-14

**Authors:** Hong Zhang, Hongmei Yang, Sanjukta Bandyopadhyay, Michael T. Milano, Chunkit Fung, Edward M. Messing, Yuhchyau Chen

**Affiliations:** 1 Department of Radiation Oncology, University of Rochester Medical Center, Rochester, NY, United States of America; 2 Department of Biostatistics and Computational Biology, University of Rochester Medical Center, Rochester, NY, United States of America; 3 Division of Hematology and Oncology, Department of Medicine, University of Rochester Medical Center, Rochester, NY, United States of America; 4 Department of Urology, University of Rochester Medical Center, Rochester, NY, United States of America; Yale University School of Medicine, UNITED STATES

## Abstract

**Introduction:**

Testicular cancer survivors (TCS) have an increased risk of additional cancers, including prostate cancer. Our understanding of the natural history of prostate cancer in testicular cancer survivors is very limited due to its rare incidence.

**Methods:**

Using the Surveillance, Epidemiology, and End Results (SEER) Registry from 1978 to 2011, we identified 282 TCS with subsequent prostate cancer and examined the tumor grade and clinical outcomes in contrast to men with primary prostate cancer in the general population.

**Results:**

TCS with a subsequent prostate cancer diagnosis were more likely to be diagnosed at a younger age than men with primary prostate cancer (65.2% vs. 37.6% for age ≤65, 34.8% vs. 62.4% for age >65, p<0.001) and were more likely to have grade III/IV tumors (46.2% vs. 37.0%, p<0.002). Longer latency between testicular and prostate cancer diagnoses was associated with a higher risk of grade III/IV (p<0.001) cancer. Despite the increased risk for high-grade tumors, 10-year prostate cancer-specific survival and overall survival were not significantly different between TCS and men with primary prostate cancer. Based on the available information in SEER, we found that prior history of radiotherapy for testicular cancer had no impact on tumor grade or survival outcomes.

**Conclusions:**

Prostate cancer in TCS was more likely to be diagnosed at a younger age and with higher grades. Risks of grade III/IV disease increased with longer latency between testicular and prostate cancer diagnoses. Radiotherapy for testicular cancer did not appear to have a significant impact on the outcome of subsequent prostate cancer.

## Introduction

Testicular cancer is a rare cancer type in American men (estimated 0.98% among all-male non-cutaneous cancers in 2021) [1], but it is the most common malignancy in young adults between the ages of 18 and 35. Testicular cancer is among the most curable solid tumors, with a 10-year overall survival rate as high as 96%. In 2017, it was estimated that there were about 269,769 men in the U.S. living with testicular cancer [2]. Because most testicular cancer patients have prolonged survival, issues associated with cancer survivorship evolve as these men age. Studies have shown an increased risk of second malignancies among testicular cancer survivors (TCS) [3–6].

Prostate cancer is the most common non-cutaneous cancer in American men (estimated 25.6% among all-male non-cutaneous cancers) [1]. Survivors of cancer treatments for testicular cancer after radiation therapy (RT) and chemotherapy have shown an increased relative risk of approximately 1.4-fold to develop subsequent prostate cancer among other second cancer types [3]. In consideration of treatment-related long-term adverse effects to the cardiovascular system and second malignancy, radiation oncologists have continued their efforts in reducing the field size of radiation treatments over the past 3 decades to exclude the supradiaphragmatic field and pelvic field. However, at the same time, there has been increased use of chemotherapy for testicular cancer treatments, leading to more concerns and recent reports focusing on chemotherapy-induced second malignancy risks [4, 7].

Because of the rarity of prostate cancer cases among TCS, the characteristics of prostate cancer in this unique population are largely unknown, posing challenges for providers caring for TCS. To examine the characteristics and clinical outcome of prostate cancer among TCS, we comprehensively reviewed data of patients with prostate cancer as a second malignancy after testicular cancer who were registered in the Surveillance, Epidemiology, and End Results (SEER) Multiple Primary database in comparison to men with a primary prostate cancer diagnosis (i.e., no prior malignancy). We also investigated if there was any effect of prior RT for testicular cancer on subsequent prostate cancer characteristics and outcomes.

## Materials and methods

### Patient database

We identified men registered in the SEER 9 registries who were diagnosed with prostate cancer at least 12 months after being diagnosed with testicular cancer between January 1, 1978 and December 31, 2011. The database was last accessed in November 2018. Eligibility criteria included the diagnosis of testicular cancer as primary cancer, age >18 years at the time of testicular cancer, and prostate cancer (with adenocarcinoma histology) as second cancer. We collected the following information recorded in SEER: age at diagnosis, year of diagnosis, grade, SEER historic stage A, survival months, COC to site recode, vital status, months since index, and radiation (S1 File).

We also identified men who were diagnosed with prostate cancer registered in the SEER during the same time period without prior cancer diagnosis to serve as a reference population. The database was last accessed in April 2018. Patients registered as "death certificate only" or "autopsy only" cases were excluded. We extracted the following information recorded in SEER: age at diagnosis, year of diagnosis, grade, SEER historic stage A, survival months, COC to site recode, and vital status (S2 File). The clinical stage was reported as local, regional, locoregional, or distant disease per SEER. Because of significant differences in outcomes between distant diseases and others, we divided the stages into two groups: distant vs. locoregional disease (including SEER classification of local, regional, and locoregional disease). Since the Gleason score was only implemented in the 1980s (64% of the study cohort had an unknown Gleason score), we categorized the tumor grade into two groups. Grade I/II was defined as well-differentiated and moderately-differentiated, and grade III/IV as poorly- and un-differentiated. PSA levels were not analyzed in this study because they were unknown for 47.4% of the cohort.

All data from the SEER database were fully anonymized before we accessed them.

### Statistical analysis

Descriptive statistics, including medians and proportions, were computed as appropriate. Chi-square tests for multinomial goodness of fit were used to evaluate the homogeneity of distributions of categorical variables (grouped by age, stage, and grade) between TCS with subsequent prostate cancer and primary prostate cancer.

Overall survival and cause-specific survival were calculated from the date of prostate cancer diagnosis to the date of death or last follow-up. Latency for prostate cancer in TCS was calculated from testicular cancer diagnosis to prostate cancer diagnosis. Cox proportional model was applied to each study group to explore the effect of age and grade on overall survival and cause-specific survival. This model was also used to assess the effects of testicular cancer radiation (RT) on overall survival and cause-specific survival. The risk of grade III/IV prostate cancer in TCS and in TCS treated with or without prior RT in reference to primary prostate cancer survivors was assessed by risk standardized morbidity ratio (SMR), with adjustment to age and era of diagnosis. To derive risk SMR, the observed number of grade III/IV (O) was divided by the number expected (E) in the primary prostate cancer population. The indirect standardized risk for TCS was computed as risk SMR multiplied by the overall crude risk in the primary prostate cancer population. The confidence interval of risk SMR was derived under lognormal distribution. Standardized risk ratio (SRR) analysis to assess RT effect on risk for grade III/IV disease was based on direct standardization, with adjustment to age and era of diagnosis. SAS procedure of STDRATE was used for risk SMR and SRR analysis. Details about the statistical methods can be found in Greenland et al. [8, 9]. Statistical significance was defined as P<0.05 (two-sided). Statistical analyses were performed using Software SAS 9.4 (SAS Institute Inc, Cary, NC) [10].

## Results

From January 1, 1978 to December 31, 2011, 282 men with prostate cancer as a second malignancy after a previous testicular cancer diagnosis were identified. The latency between the two diagnoses ranged from 12 to 443 months, with a median of 211 months. For the reference population, 893,703 men were identified in SEER with prostate cancer as a primary diagnosis during the same time period. Table 1 shows the characteristics of prostate cancer in TCS and primary prostate cancer identified in the SEER database.

**Table 1:** Study characteristics.

	Prostate Cancer in TCS		Primary Prostate Cancer	
	Number	%	Number	%
**All**	282		893703	
**Age, in years (p <0.001)**				
**≤ 65**	184	65.2	336221	37.6
**> 65**	98	34.8	557482	62.4
**Stage**^**1**^ **(p = 0.262)**				
**Localized/regional**	210	96.8	612810	95.1
**Distant**	7	3.2	31324	4.9
**Grade**^**2**^ **(p = 0.002)**				
**Grade I/II**	143	53.8	519142	63.0
**Grade III/IV**	123	46.2	305493	37.0

1. Stage is unknown for 65 TCS and 249569 primary prostate cancer patients.

2. Grade is unknown for 16 TCS and 69068 primary prostate cancer patients.

Among the 282 TCS with prostate cancer, 65.2% were ≤ 65 years of age, with a median age of 61. In comparison, among men with prostate cancer as a primary diagnosis, 37.6% were ≤65 years of age, with a median age of 69 (p<0.001; Table 1).

There was no statistically significant difference in prostate cancer stage distribution between the two cohorts (p = 0.262; Table 1). In contrast, 46.2% of TCS with prostate cancer had grade III/IV disease, versus 37.0% in men with prostate cancer as a primary diagnosis (p = 0.002; Table 1).

**Fig 1:**
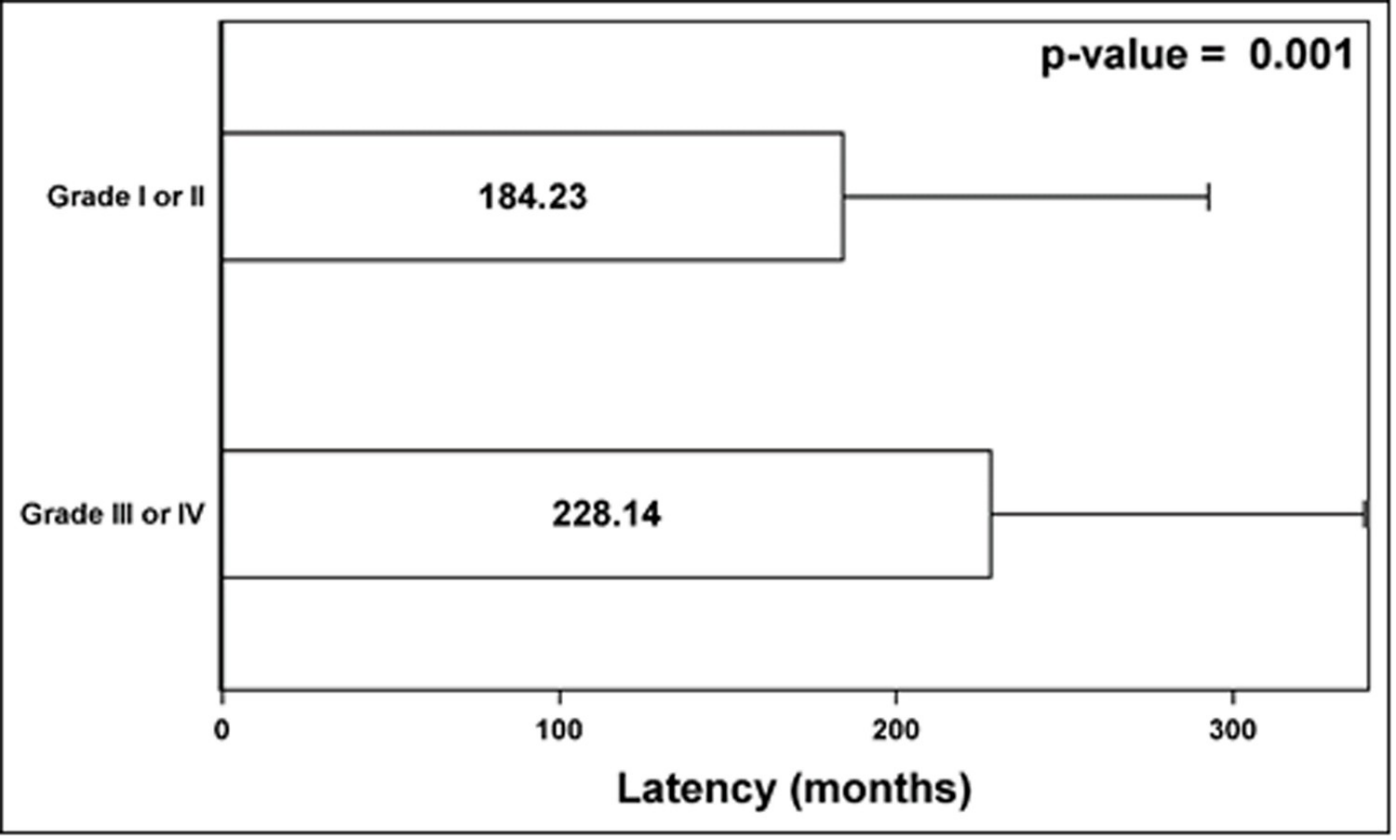
Relationship between latency and prostate cancer grade in testicular cancer survivors.

A longer latency from the initial diagnosis of testicular cancer to the diagnosis of prostate cancer was associated with a higher incidence of grade III/IV disease (median latency in months for grade I/II and grade III/IV were 184.2 and 228.1 respectively, p = 0.001; Fig 1).

**Fig 2:**
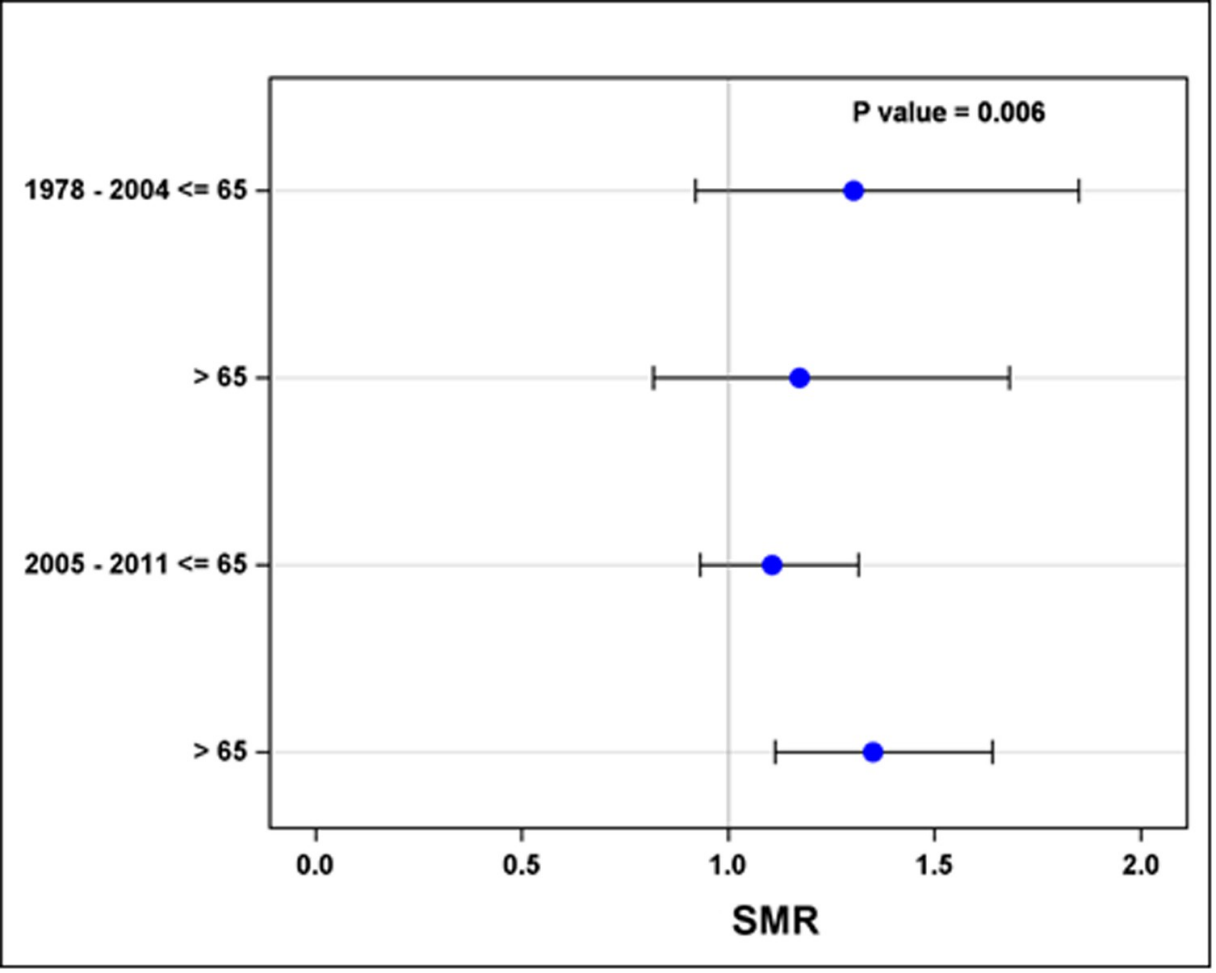
The risk standardized morbidity ratio (SMR) of prostate cancer grade in TCS with the stratification by age and era of diagnosis compared with primary prostate cancer survivors.

While controlling for age and era of diagnosis, the risk SMR analysis showed a significantly increased risk for grade III/IV disease among TCS, compared with men with primary prostate cancer (Fig 2, p = 0.006).

**Fig 3:**
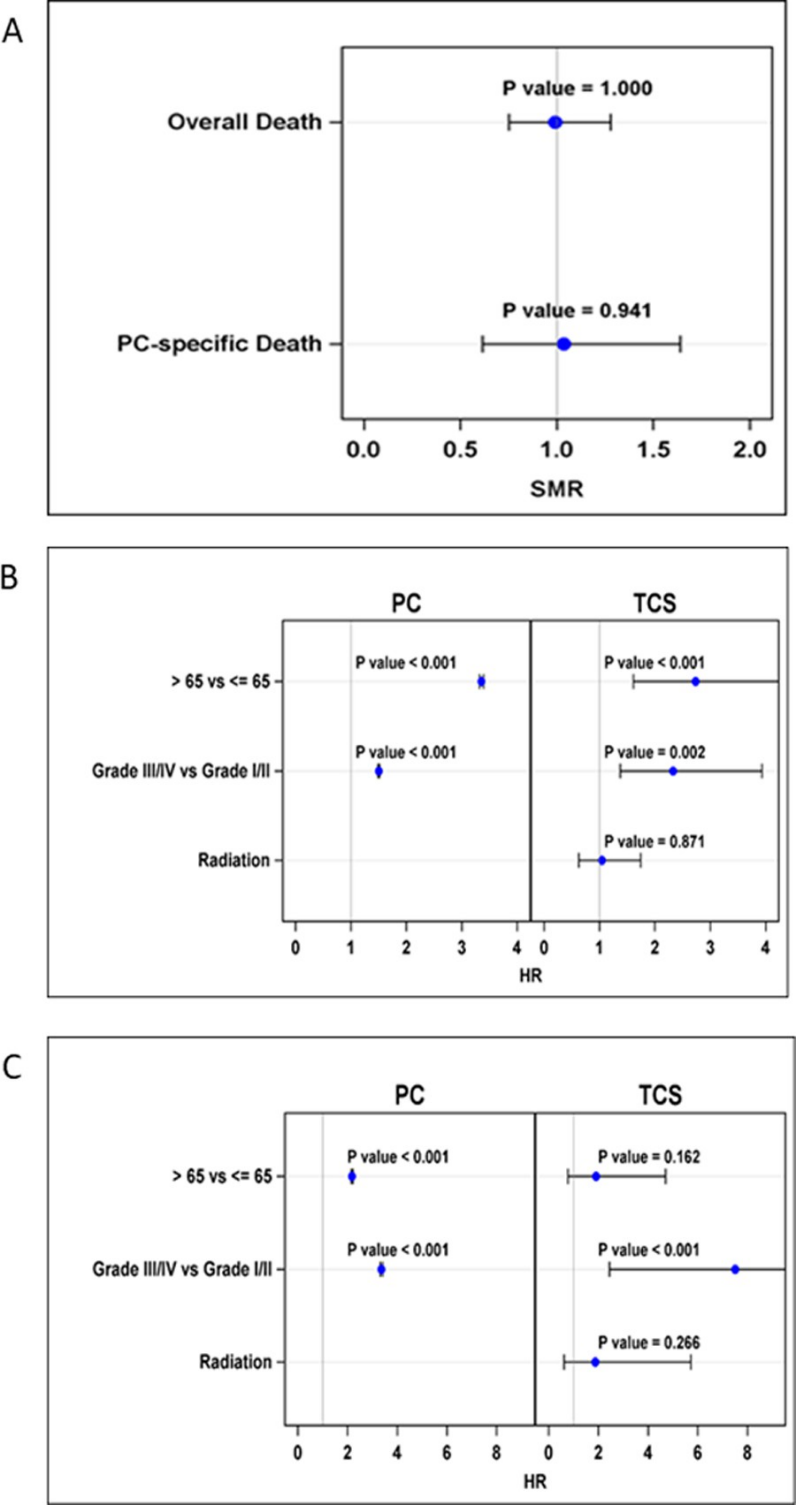
Outcomes for TCS and men with primary prostate cancer. A. Ten-year age- and grade-adjusted overall and prostate cancer-specific standardized mortality ratio (SMR) for TCS vs. primary prostate cancer; B. Hazard ratio of ten-year overall survival; C. Hazard ratio of ten-year prostate cancer-specific survival.

**Fig 4:**
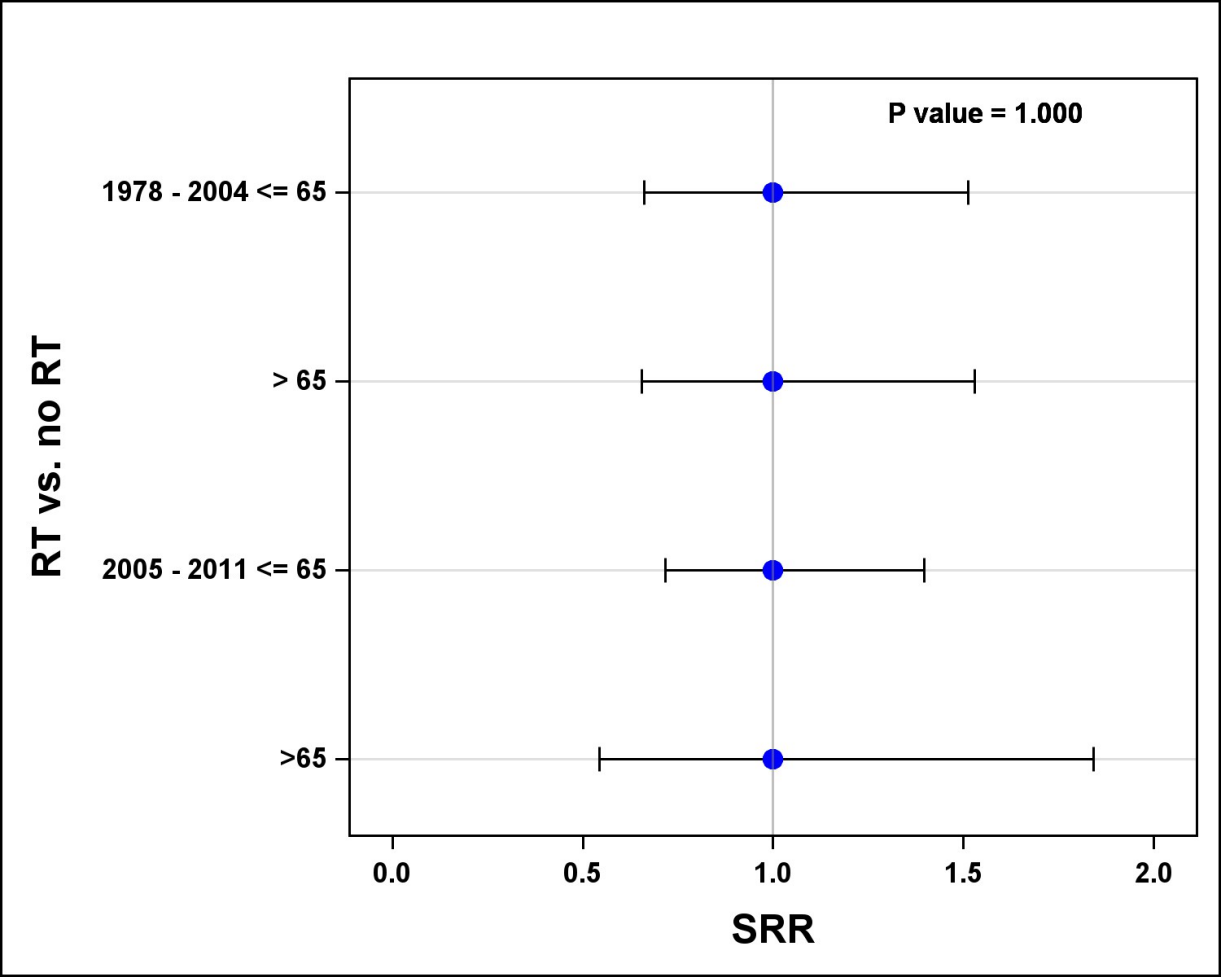
Effect of prior RT for testicular cancer on prostate cancer grades: The SRR for grade III/IV prostate cancer with adjustment to age and era of diagnosis.

Only three (1.1%) TCS died from testicular cancer. Ten-year age- and grade-adjusted SMR for TCS vs. men with primary prostate cancer showed no statistical differences in overall death rate and prostate cancer-specific death rate (Fig 3A). The Cox proportional hazard model showed that age and grade were significantly associated with overall survival and prostate cancer-specific survival for TCS with second prostate cancer and men with primary prostate cancer. Men >65 years of age, as well as men with high-grade disease (grade III/IV), had worse ten-year overall and prostate cancer-specific survival when comparing with men ≤65 or men with low grade (grade I/II) diseases (Fig 3B). Furthermore, prior RT for TCS had no significant effect on 10-year overall survival or prostate cancer-specific survival (Fig 3C). When controlling for age at the time of diagnosis and era of diagnosis of prostate cancer, the SRR analysis showed no statistically significant increased risk for grade III/IV disease among TCS who had received prior RT, comparing with TCS with no prior RT (Fig 4).

## Discussion

Our study examined data from the SEER database to characterize clinical and pathological features of prostate cancer in TCS. To our knowledge, our study is the largest and most comprehensive investigation of this particular cancer population. Testicular cancer affects young adults at their prime age, and though it has a high cure rate, survivors are left with a lifetime risk of second cancer. Thus, the outcome of our investigation provides much-needed information for testicular cancer survivors should they face a prostate cancer diagnosis later in life. We observed that testicular cancer survivors were diagnosed with prostate cancer at a younger age. They also had an increased risk of high-grade disease, especially with the longer latency between testicular and prostate cancer diagnoses. These findings underscore the importance of early prostate cancer screening for testicular cancer survivors. Of note, our analyses revealed that 65.2% of prostate cancer in TCS was diagnosed in patients younger than 65 years of age but only 37.6% in the general population. Therefore, based on our observation, testicular cancer survivors may benefit from early prostate cancer screening.

While the SEER database is very useful for cancer epidemiology studies of rare diseases, we acknowledge that there are many limitations using data from a large cancer registry. The known weaknesses include missing or incomplete data, lack of therapy information such as drug or chemotherapy treatments, lack of details regarding radiation treatments (such as RT dose and the volume), and missing laboratory data such as testosterone level of patients with prostate cancer. All of these would have offered valuable information about our study population. Nevertheless, only through this type of large longitudinal database of a cancer registry is it possible to examine rare and unique cancer populations and compare the findings to the general population in the same cancer registry for the same period of time.

We observed that the median age of prostate cancer diagnosis among TCS was eight years younger than that of the reference population of men diagnosed with primary prostate cancer. Although the exact reason for this observation is not clear, this difference may be multifactorial, including cancer susceptibility at a younger age in TCS and/or the result of earlier detection under routine cancer surveillance as part of survivorship care after testicular cancer treatments. The difference in PSA screen-detected prostate cancer rates between the two study cohorts, unknown to us, may also have an impact on the age of prostate cancer diagnosis. One other confounding factor is that men diagnosed with testicular cancer in earlier years might be less likely to live long enough to have prostate cancer diagnosed at an older age. For example, age-standardized 10-year net survival of testicular cancer in England and Wales was 69.2% from 1970 to 1971, 83.2% from 1980 to 1981, 91.8% in 1990 to 1991, 96.1% from 2000 to 2001, and 98.2% from 2010 to 2011 [11]. In our study cohorts, 52.1% of prostate cancer cases among TCS were diagnosed between 1978 and 2004, comparing with 63.2% of prostate cancer cases among men with primary prostate cancer in the same period (p<0.01). Therefore, this confounding factor may influence the age of prostate cancer diagnosis in TCS.

We observed 46.2% prostate cancer among TCS with grade III/IV disease, but 37.0% among men with primary prostate cancer. The significance of this observation remains unclear for now as we did not observe a statistically significant difference in ten-year prostate cancer-specific or overall survival in TCS compared with men with prostate cancer as the primary diagnosis, with adjustment of age and era of diagnosis. The long natural history of localized prostate cancer and limited death events among TCS (only 23/282 TCS died from prostate cancer) might have limited the power to detect any statistically significant differences in survival outcomes.

Some studies have indicated that the risk of second cancers among TCS is related, at least in part, to the treatment modality used for testicular cancer, specifically radiotherapy [3, 12]. We found that prior radiotherapy for testicular cancer did not increase the risk of developing high-grade prostate cancer compared with no prior radiotherapy. We acknowledge that our observation might be limited by the small sample size as there were only 74/282 TCS (26%) treated with radiotherapy in this cohort. While the SEER registry did not provide information on chemotherapy and details of radiotherapy, the RT doses for testicular cancer had been relatively low in the 20 Gy to 30 Gy range without much variation. It is also known that radiotherapy volume has been reduced to minimize late effects since 2005, especially after the publication of landmark randomized trials. These studies showed no survival benefit with higher doses or larger RT fields (i.e., the inclusion of ipsilateral iliac nodal regions in addition to para-aortic lymphatics) [13, 14]. Further investigation using another database may be helpful to support or dispute our findings.

It is interesting to note that longer latency between testicular cancer and prostate cancer diagnoses was associated with a higher risk of grade III/IV prostate cancer. While our observation from the SEER database does not allow the investigation of the cause or mechanism of this observation, other observational studies have shown that older men are more likely to have prostate cancer with higher risk features [15, 16]. The underlying reasons for this trend may be multifactorial, including the possible lack of prostate cancer screening among the elderly. Our finding does support the importance of early and vigilant prostate cancer screening for TCS.

The impact of circulating testosterone on the risk of prostate cancer has been controversial [17]. A relevant point of discussion for the TCS population with subsequent prostate cancer is the potential influence of testosterone levels after orchiectomy. TCS are at higher risk of hypogonadism [18–21], and a prior study reported that up to 15% of TCS had subnormal testosterone levels or used androgen replacement [20]. Because only up to 15% TCS might have altered testosterone levels, the impact on our study population would not have been significant. Nevertheless, testosterone levels are important correlative data and would have been informative if the SEER database had contained this laboratory information.

## Conclusion

Using the SEER registry, we found that prostate cancers in TCS were more likely diagnosed at a younger age and had a higher risk of being grade III/IV disease. Longer latency between testicular and prostate cancer diagnoses was associated with an increased risk of high-grade tumors. Prior history of radiotherapy for testicular cancer did not appear to increase the risk of developing grade III/IV prostate cancer compared with no prior radiotherapy. These findings provide important considerations when caring for TCS.

## Supporting information

S1 File Dataset of TCS with prostate cancer. (XLSX)

S2 File Dataset of primary prostate cancer. (XLSX)
